# Follow up of utility and value of totally implantable chemotherapy catheter in 233 brazilian patients receiving chemotherapy to treat cancer

**DOI:** 10.1590/0100-6991e-20233367-en

**Published:** 2023-06-22

**Authors:** RODRIGO MELO CÉSAR, ANA P. DRUMMOND LAGE, ALBERTO WAINSTEIN

**Affiliations:** 1 - Faculdade Ciências Médicas de Minas Gerais, Pós Graduação - Belo Horizonte - MG - Brasil

**Keywords:** Catheters, Antineoplastic Agents, Patient Satisfaction, Obstrução do Cateter, Antineoplásicos, Satisfação do Paciente

## Abstract

**Objectives:**

the present study aims to evaluate cancer patients related to the catheter flow and the general satisfaction of these patients.

**Methods:**

we studied 233 individuals diagnosed with cancer who underwent chemotherapy treatment through venous access through portocath between January 2015 and December 2019.

**Results:**

97% of the patients consulted had palliative chemotherapy, and 99.1% of patients reported satisfaction with the implantation process and treatment method. Regarding catheter flow according to venous return and drip during drug infusion, 98.7% of individuals presented good flow.

**Conclusions:**

the results show that catheter flow was satisfactory in all implant sites observed and emphasize the advantages of using a totally implanted catheter. This benefice happens due to the reduction of emotional factors that cause stress in cancer patients receiving chemotherapy, as well as the reduction of trauma and discomfort experienced by patients during the infusion of peripheral chemotherapy.

## RESUMO

**Objetivos:**

avaliar pacientes com câncer em relação ao fluxo do cateter e a satisfação geral desses pacientes.

**Métodos:**

estudo retrospectivo avaliando prontuários de 233 indivíduos com diagnóstico de câncer que realizaram tratamento quimioterápico por acesso venoso por portocath entre janeiro de 2015 e dezembro de 2019.

**Resultados:**

97% dos pacientes realizaram quimioterapia paliativa, e 99,1% dos pacientes relataram satisfação com o processo de implantação e método de tratamento. Em relação ao fluxo do cateter de acordo com o retorno venoso e gotejamento durante a infusão da droga, 98,7% dos indivíduos apresentaram bom fluxo.

**Conclusões:**

os resultados mostram que o fluxo do cateter foi satisfatório em todos os locais de implante observados e enfatizam as vantagens do uso de um cateter totalmente implantado. Esse benefício acontece devido à redução de fatores emocionais que causam estresse em pacientes oncológicos em tratamento quimioterápico, bem como a redução de traumas e desconfortos vivenciados pelos pacientes durante a infusão de quimioterápicos periféricos.

## INTRODUCTION

Cancer is a worldwide public health problem, 625,000 new cases being expected in Brazil between 2020 and 2022, according to data from the National Cancer Institute (INCA). Chemotherapy is a treatment with intravenous medication and, therefore, catheters are essential in most procedures. A fully implantable venous catheter (FIVC) model with a subcutaneous chamber was developed in the 1970s, and has evolved since then. Totally implantable catheters reduce the risk of local problems and provide greater comfort to the patient, through continuous infusion in a home environment. There are different types of catheter, which can be implanted in different vessels according to the characteristics of the prescribed chemotherapy protocol, duration of treatment, integrity of the venous network, and patient preferences^1^.

In cancer patients undergoing chemotherapy, the preferable venous access is central, since many antineoplastic drugs are notoriously vesicant. Many oncology units still deliver chemotherapy primarily via the peripheral route, yet there is consensus in the medical community that the infusion of vesicant drugs into a peripheral vein is potentially dangerous, associated with a high risk of extravasation, infiltration, phlebitis, local tissue damage, and progressive decrease in available peripheral veins^1^. The National Supplementary Health Agency (ANS) standards for infusion therapy recommend central venous access (including peripherally inserted central catheters - PICC) for bolus administration of vesicant medications; if a peripheral access is chosen, a new access location must be used for each administration, and the site must be documented to avoid repeated use. However, continuous infusion of vesicants must be performed exclusively through a central route^1^.

Another model of long-term catheter is the totally implantable one, known as Port-A-Cath. It is a catheter with a diameter of less than 10 Fr, which can be implanted through a peripheral or central vein and, after passing through the subcutaneous route, is connected to a reservoir usually implanted over the muscle fascia of the site chosen for making the pocket. As no segment of the set is externalized, this type of catheter has a lower risk of infection and greater durability compared with semi-implantable ones. The main indications for the placement of totally implantable catheters are the need for frequent venous access, use of vesicant drugs and inadequate peripheral venous system^1^.

PICC and Port-A-Cath can greatly improve patient quality of life and reduce nurses’ workload. Port-A-Cath is best suited for patients who require long-term, high-dose chemotherapy, such as eight cycles or more, and those who live far from the hospital, while PICC is best suited for patients with chemotherapy of shorter durations, such as four-cycle regimes, or living close to the hospital^8^.

PICCs are associated with a higher risk of related deep vein thrombosis and other adverse events when compared with Port-A-Cath. This risk must be considered when choosing a vascular access device for chemotherapy, especially in patients with solid neoplasias^9^.

Complications with Port-A-Cath may be related to the implantation technique, handling, and maintenance for drug administration. Possible complications involve risk of infection, venous thrombosis, fracture, catheter embolization, and low catheter flow^4^.

The fully implanted catheters are composed of a stainless steel or titanium reservoir, a siliconized catheter, and a central septum. This septum is covered by a self-sealing diaphragm capable of receiving 1,000 to 2,000 needle punctures. Its access is made through puncture with Huber-type needles, which have a special non-fragmenting bevel. Port-A-Cath catheters guarantee a highly reliable and practical venous access and have been widely used in several studies carried out in the field of medicine^2^.

A study with 281 patients undergoing chemotherapy treatment using Port-A-Cath showed a complication rate of 26%, with thrombosis occurring in 16.4% of cases, infection in 3.2%, malpositioning in 3.2%, and pneumothorax in 1.4%. Complications are not associated with the site of catheter implantation, and the chosen vessel is not associated with more severe or frequent complications^5^.

However, some studies report that Port-A-Cath devices implanted in the arms, regardless of the side, have a higher risk of thrombosis than those implanted in the thorax.

Catheters in the arm occupy a large proportion of the intravascular lumen as they are in the peripheral veins. Repetitive arm movements can also contribute to the incidence of thrombosis. In addition, arm Port-A-Caths require a longer intravenous portion than chest wall ones. Therefore, prolonged contact between the catheter and the intravascular wall can result in endothelial damage, reduced blood flow, and consequent thrombosis^7^.

The focus of this work is to evaluate aspects of the use of a fully implantable long-term Port-A-Cath, known as Portocath, implanted through a peripheral or central vein and connected to a reservoir positioned on the muscular fascia of the chosen location. It is not uncommon for the surgeon who implants the catheter not to accompany the patient after the surgical procedure, except in the case of immediate complications, and consequently not being aware of the benefits the procedure has brought to the patient. Therefore, it is important to seek a better understanding of the use of the fully implantable, long-term catheter in cancer treatment.

It should be noted that, despite the standardization and widespread use of Central Venous Catheters (CVCs) in different types of therapy and invasive monitoring in Brazil, few related studies are found in the country.

Thus, this research proposes to detail the epidemiological and clinical profile of cancer patients with CVC, to investigate adverse events resulting from treatment through central venous access, and to assess the perception of Quality of Life of patients with this treatment method.

## METHODS

This study has a descriptive character. The research was carried out in an oncology center in the Metropolitan Region of Belo Horizonte, with units in the cities of Betim, Contagem, and Belo Horizonte. The service assists patients in the scope of supplementary health (insurance-covered).

We selected a convenience sample with 233 patients from the three service units, who started chemotherapy treatment through venous access using Portocath, between January 2015 and December 2019.

Inclusion criteria involve patients from the Unified Public Health System (SUS) and the supplementary health one undergoing a Port-A-Cath implant for chemotherapy treatment in the considered period. Exclusion criteria were patients whose registration regarding the catheter insertion was incomplete, since the sampling was carried out based on documentary and retrospective data collection.

Data were collected by the nurse responsible for the chemotherapy catheters in the institution’s database, using an instrument developed specifically for this study, called ‘Chemotherapy Catheter Questionnaire’. This questionnaire’s sources of data were the electronic medical records accessed through the SpData Software. We accessed data on medical evolution and prescription, catheter implantation, anatomopathological reports, evolution of the nurse’s consultation, evolution of the pharmacist, and personal data. The information collected in the database can be categorized as follows:

- Sociodemographic: sex, date of birth, medical record number, profession, skin color, education level, and municipalities of residence.
- Clinical: comorbidities, medical history, alcoholism, smoking, sedentary lifestyle, body mass index, histological type of tumor, type of chemotherapy (neoadjuvant, adjuvant, or palliative), previous treatments, drugs used in current chemotherapy, duration of treatment (number of cycles), number of total punctures per month, and patient outcome.
- Catheter: indication, insertion site with laterality, drug flow, catheter thrombosis, ease of puncture and moment of chemotherapy when catheter placement was indicated, waiting time between indication and implantation, previous central accesses, time between implantation and first infusion, and other uses, such as hydration, non-chemotherapy medications, or blood collection.
- Complications: local or systemic complications, date of occurrence, duration, severity, and adopted measures.

As this is a retrospective analysis of medical records in the last five years, it was not possible to locate all research subjects or legal guardians for the informed consent process, due to the estimated sample size, as well as due to the possibility of analyzing medical records of patients who died. For these reasons, we requested the waiver of the Informed Consent Form.

The Medical Sciences Research Ethics Committee - MG/ (CEPCM-MG) approved the work on 09/08/2021, with registration CAAE 36695420.1.0000.5134.

## RESULTS

The mean sample age was 59.8 years, ranging from 18 to 94, and the distribution by sex was 65.7% for females (154 individuals) and 34.3% for males (80 individuals), as shown in Table 1. Of this group of patients, six were undergoing adjuvant chemotherapy (2.6%), one was undergoing neoadjuvant chemotherapy (0.4%), and 227, palliative (97%).

**Table 1:** Demographic data of patients with FIVC implantation.

Variables	n	Mean	SD	Min.	Max.	Median
Age	84	59.7	13.7	27	90	60.5
Weight (kg)	84	72.9	15.0	40.8	136.0	70.9
Height (m)	82	1.64	0.10	1.45	1.88	1.63
BMI	82	27.2	4.6	16.5	38.5	26.9
C1 Medication - Expected Cycles	85	10	7	1	36	10
C2 Medications - Expected Cycles	62	11	8	1	46	12
C3 Medications - Expected Cycles	33	10	5	1	24	12

Clinical indications for FIVC implantation included long treatment period, poor peripheral access, greater convenience, risk of extravasation, or multiple indications.

The site of catheter implantation was, in most cases, the right subclavian vein, with 132 patients (56.7%); 20 patients (8.6%) had the catheter implanted in the left subclavian vein, 67 (28.7%) in the right jugular, 13 (5.5%) in the left jugular, and one (0.5%) in the left brachial vein, as described in Table 2.

**Table 2:** Access routes used for FIVC implantation

Catheter Location	Qty. patients	%
Right subclavian vein	130	55.8%
Right jugular vein	46	19.7%
Right internal jugular vein	21	9.0%
Left subclavian vein	20	8.6%
Left jugular vein	7	3.0%
Left internal jugular vein	6	2.6%
Left brachial vein	1	0.4%
Subclavian vein	1	0.4%
Subclavian vein	1	0.4%
Total	233	100.0%

Patient satisfaction was defined as the possibility of ending treatment without the need for new venous access and easy punctures. Regarding satisfaction with the chemotherapy catheter, 231 patients reported good satisfaction (99.1%) and two reported moderate satisfaction (0.9%). No patient reported dissatisfaction or regret for having implanted the catheter. Patients who did not regret having implanted the catheter and did not complain about events related to its use were considered satisfied.

We evaluated catheter flow according to venous return and dripping during drug infusion: 230 subjects had good flow (98.7%), two patients had moderate flow (0.9%), and one, poor flow (0.4%).

## DISCUSSION

Since their introduction in 1980, FIVC have been widely used for chemotherapy, especially in patients with poor peripheral venous circulation, mainly in the context of palliative chemotherapy, with prolonged treatment6. Initially, they had little use in Brazil, but after its incorporation into the SUS, it became a large volume procedure in all hospitals that treat cancer patients.

The fully implanted, long-term venous catheter is a practical venous access, with few risks for the patient’s treatment, and which favors the quality of life of cancer patients, since these catheters practically do not restrict physical mobility, allow greater freedom in choosing their activities, and favor body image2. The immediate complications of the catheter, such as pneumothorax, bruises, and hemorrhages have always attracted the attention of surgeons. Although potentially serious, they are rare and tend to become less and less prevalent with greater staff training, better catheters, and more appropriate surgical conditions, such as the use of image intensifiers and perioperative ultrasound.

According to the completed questionnaires, practically all patients monitored had a good flow in the catheter and good satisfaction with it (only two had a moderate flow and one had a poor flow), which can be considered excellent when compared with data in the literature^2^. Given that catheter flow is naturally almost always good, it is not possible to relate cases of poor flow quality to any other variable queried by the survey instrument (age, sex, catheter site,…). Therefore, poor flow in the catheter does not depend on the insertion site, but on other factors not addressed in the used questionnaire. No patient had treatment delayed or interrupted due to lack of venous access.

Given this practically unanimous behavior of catheter satisfaction, it is not possible to relate the cases of dissatisfaction with any other variable answered in the questionnaires (age, sex, catheter site,…). Therefore, dissartisfaction with the catheter is due to individual patient factors, not collected in the questionnaire and not detectable in statistical tests.

We believe that our results confirm that infusion through the chemotherapy catheter has positive aspects, such as greater safety in receiving treatment, a decrease in the number of punctures received, reduction of pain and stress during infusion, and especially greater adherence to treatment, without interruption of cycles per lack of venous access. The advantages of using a fully implanted catheter are highlighted in view of the reduction of emotional factors that cause stress in cancer patients receiving chemotherapy, as well as the reduction of trauma and discomfort experienced by patients during the infusion of peripheral chemotherapy. 

We believe that the results were good, since most of the patients were receiving palliative treatment and required a long treatment period and several punctures.

In addition, only one catheter was placed in the arm, a site with a higher complication rate than the thorax, corroborating the good results.

## CONCLUSION

The fully implantable catheter has benefited cancer patients who, during their treatment, may need prolonged intravenous chemotherapy, allowing treatment for long periods without the need for new venous accesses. This study presented an evaluation of the effect of using a fully implanted catheter on the infusion flow during chemotherapy treatment and a survey of the epidemiological data of the patients, as well as the general satisfaction of the patients with the treatment method, demonstrating good results in the vast majority with 99.1% of patients satisfied during the chemotherapy period. These results and conclusions are of great importance for the surgeon who implants the catheter and is often unaware of how beneficial his procedure was for the patient’s life.

## References

[1] Zerati AE, Wolosker N, de Luccia N, Puech-Leão P (2017). Cateteres venosos totalmente implantáveis histórico, técnica de implante e complicações. J Vasc Bras.

[2] Tang T, Liu L, Li C, Li Y, Zhou T, Li H Which is better for patients with breast cancer: totally implanted vascular access devices (TIVAD) or peripherally inserted central catheter (PICC). World J Surg.

[3] Taxbro K, Hammarskj F, Thelin B, Lewin F, Hagman H, Hanberger H (2019). Clinical impact of peripherally inserted central catheters vs implanted port catheters in patients with cancer an open-label, randomised, two-centre trial. Br J Anaesth.

[4] Gallieni M, Pittiruti M, Biffi R (2008). Vascular access in oncology patients. CA Cancer J Clin.

[5] Martins FTM, Carvalho EC (2008). Patients' perception regarding the use of a long-term catheter. Rev Esc Enferm USP.

[6] El-Balat A (2018). Catheter-related complications of subcutaneous implantable venous access devices in breast cancer patients. in vivo.

[7] Okazaki M, Oyama K, Kinoshita J, Miyashita T, Tajima H, Takamura H (2019). Incidence of and risk factors for totally implantable vascular access device complications in patients with gastric cancer A retrospective analysis. Mol Clin Oncol.

[8] Miranda RBD, Lopes JRA, Cavalcante RN, Kafejian O (2008). Perviedade e complicações no seguimento de cateteres venosos totalmente implantáveis para quimioterapia J. vasc. Bras.

[9] Rodrigues CC, Guilherme C, Costa MLD, Carvalho ECD (2012). Fatores de risco para trauma vascular durante a quimioterapia antineoplásica contribuições do emprego do risco relativo. Acta Pau Enferm.

